# Spatial and Temporal Distribution of *Mycobacterium tuberculosis* Complex Infection in Eurasian Badger (*Meles meles*) and Cattle in Asturias, Spain

**DOI:** 10.3390/ani11051294

**Published:** 2021-04-30

**Authors:** Cristina Blanco Vázquez, Thiago Doria Barral, Beatriz Romero, Manuel Queipo, Isabel Merediz, Pablo Quirós, José Ángel Armenteros, Ramón Juste, Lucas Domínguez, Mercedes Domínguez, Rosa Casais, Ana Balseiro

**Affiliations:** 1 Servicio Regional de Investigación y Desarrollo Agroalimentario del Principado de Asturias (SERIDA), 33300 Villaviciosa, Spain; cristina.blancovazquez@serida.org (C.B.V.); rosacg@serida.org (R.C.); 2 Laboratório de Imunologia e Biologia Molecular, Instituto de Ciências da Saúde, Universidade Federal da Bahia, 40.110-100 Salvador, Bahia, Brazil; tadbarral@gmail.com; 3 Centro de Vigilancia Sanitaria Veterinaria VISAVET, Universidad Complutense, 28040 Madrid, Spain; bromerom@visavet.ucm.es (B.R.); lucasdo@visavet.ucm.es (L.D.); 4 Servicio de Sanidad y Producción Animal del Principado de Asturias, 33007 Oviedo, Asturias, Spain; manuelantonio.queiporodriguez@asturias.org; 5 Laboratorio Regional de Sanidad Animal del Principado de Asturias, 33201 Gijón, Asturias, Spain; isabel.meredizgutierrez@asturias.org; 6 Dirección General del Medio Natural y Planificación Rural del Principado de Asturias, 33007 Oviedo, Asturias, Spain; pablo.quirosmenendezdeluarca@asturias.org (P.Q.); joseangel.armenterossantos@asturias.org (J.Á.A.); 7 Animal Health Department, NEIKER-Instituto Vasco de Investigación y Desarrollo Agrario, 48160 Derio, Bizkaia, Spain; rjuste@neiker.eus; 8 Departamento de Sanidad Animal, Facultad de Veterinaria, Universidad Complutense, 28040 Madrid, Spain; 9 Unidad de Inmunología Microbiana, Centro Nacional de Microbiología, Instituto de Salud Carlos III, 28029 Madrid, Spain; mdominguez@isciii.es; 10 Departamento de Sanidad Animal, Facultad de Veterinaria, Universidad de León, 24071 León, Spain; 11 Departamento de Sanidad Animal, Instituto de Ganadería de Montaña (CSIC-Universidad de León), Finca Marzanas, Grulleros, 24346 León, Spain

**Keywords:** *Meles meles*, badger, tuberculosis, *Mycobacterium tuberculosis* complex, P22 ELISA, isolation, serology, cattle, Atlantic Spain

## Abstract

**Simple Summary:**

The aim of the present work was to investigate the prevalence, spatial distribution, and temporal distribution of tuberculosis in 673 free-ranging Eurasian badgers (*Meles meles*) and cattle from Asturias (Atlantic Spain) during a 13-year follow-up. The study objective was to assess the role of badgers as a reservoir of tuberculosis for cattle and other sympatric wild species in the region. During the follow-up, 27/639 badgers (4.23%) were positive for the *Mycobacterium tuberculosis* complex based on bacterial isolation, while 160/673 (23.77%) were positive based on P22 ELISA. Badger infection was spatially and temporally associated with cattle herd infection.

**Abstract:**

The present work investigated the prevalence, spatial distribution, and temporal distribution of tuberculosis (TB) in free-ranging Eurasian badgers (*Meles meles*) and cattle in Asturias (Atlantic Spain) during a 13-year follow-up. The study objective was to assess the role of badgers as a TB reservoir for cattle and other sympatric wild species in the region. Between 2008 and 2020, 673 badgers (98 trapped and 575 killed in road traffic accidents) in Asturias were necropsied, and their tissue samples were cultured for the *Mycobacterium tuberculosis* complex (MTC) isolation. Serum samples were tested in an in-house indirect P22 ELISA to detect antibodies against the MTC. In parallel, data on MTC isolation and single intradermal tuberculin test results were extracted for cattle that were tested and culled as part of the Spanish National Program for the Eradication of Bovine TB. A total of 27/639 badgers (4.23%) were positive for MTC based on bacterial isolation, while 160/673 badgers (23.77%) were found to be positive with the P22 ELISA. The rate of seropositivity was higher among adult badgers than subadults. Badger TB status was spatially and temporally associated with cattle TB status. Our results cannot determine the direction of possible interspecies transmission, but they are consistent with the idea that the two hosts may exert infection pressure on each other. This study highlights the importance of the wildlife monitoring of infection and disease during epidemiological interventions in order to optimize outcomes.

## 1. Introduction

Animal tuberculosis (TB) is caused by infection with members of the *Mycobacterium tuberculosis* complex (MTC), mainly *M. bovis* and, to a lesser extent, *M. caprae*. TB is a zoonotic disease that subjects livestock worldwide and can cause substantial economic losses [1]. Its main domestic reservoir is cattle, and eradication campaigns spanning more than four decades in developed countries including Spain have been quite but not totally successful [2]. Wildlife hosts are also susceptible to *M. bovis* and can act as reservoirs of the infection for livestock. Badgers (*Meles meles*) and wild boars (*Sus scrofa*) are major wildlife reservoirs of *M. bovis* for bovine TB in several European countries. *M. bovis*-infected badgers have been found in Ireland [3], the United Kingdom (UK) [4], France [5,6], and Spain [7,8]; in Ireland and the UK, badgers are recognized as major reservoirs with the potential to transmit infection to local cattle herds [9,10]. Infection in wild boars has been described in France and Italy, as well as the southern and central Iberian Peninsula [5,11].

In Spain, the southern and central regions, which have a Mediterranean climate, show a TB cattle herd-prevalence as high as 12.3%, while the northwestern region with an Atlantic climate shows an overall prevalence of 0.02–0.08% but with hotspots where prevalence can be as high as 5% [2,12]. This variability may reflect several factors. One is climate: drier, hotter areas in the south favor animal aggregation and therefore disease spread [13]. Another factor is type of cattle: dairy herds, in which TB prevalence is lower, concentrate in the north, while beef and bullfighting cattle are more abundant in the central and southern regions [13]. A third factor is cattle management: beef and bullfighting cattle herds are extensively managed with a lower biosecurity and are therefore at higher risk of infection due to contact with animals in other herds, as well as with other potentially infected domestic (goat/sheep) or wildlife species [14,15,16].

Recent studies have suggested that badgers may be a reservoir of *M. bovis* infection in hotspots in Atlantic Spain, as reported in Ireland and the UK [12,17]. In addition, wild boar, red deer (*Cervus elaphus*), and, to a lesser extent, fallow deer (*Dama dama*) are major wildlife reservoirs in continental Mediterranean habitats, where wild boars can show a TB prevalence of >50% [14]. In contrast, wild boars in Atlantic habitats show a TB prevalence of around only 5% [16]. Mid- and long-term studies of wildlife reservoirs such as badgers and wild boar can shed light on the spatial and temporal dynamics of TB, as well as on risk to local livestock [14,18,19].

Along these lines, the present work investigated the prevalence, spatial distribution, and temporal distribution of TB in free-ranging Eurasian badgers and cattle from Asturias (Atlantic Spain) during a 13-year follow-up. The study objective was to assess the potential role of badgers as a TB reservoir for cattle and other sympatric wild species such as wild boar in the region.

## 2. Materials and Methods

### 2.1. Ethics Statement

All methods were carried out in accordance with relevant guidelines and regulations. All experimental procedures with trapped badgers were approved by the Government of the Principality of Asturias (010/07-01-2011, PROAE 20/2015, PROAE 47/2018).

### 2.2. Study Area

The study was carried out in the region of Asturias in northwestern Spain. This region has an Atlantic climate, and the temperature ranges from −4 to 8 °C in the coldest months; precipitation is abundant throughout the year, annually reaching 1400–2100 mm [20]. More than 30% of the territory is forest and mainly comprises oaks, beech, and birch woods. Asturias is bordered to the north by the Cantabrian Sea and to the south by the Cantabrian Range. Nowadays, the cattle population is 391,797 animals and 15,856 herds, and the well-studied badger population shows intermediate density of 3.81 adults/km^2^ [21].

### 2.3. Description of TB Detection in Badgers and Cattle in Asturias, 2008–2020

#### 2.3.1. Badgers

From 2008 to 2020, 673 badgers from Asturias were necropsied (see Supplementary Material 1: Raw Data); these animals came from western (*n* = 77), central (*n* = 220), and eastern (*n* = 376) parts of Asturias. Of the 673 animals, 98 were trapped badgers that had been captured from setts located close to TB cattle herds and subsequently euthanized; these 98 animals came nearly equally from the three areas. Badgers were captured in steel mesh box traps baited with peanuts and anesthetized with ketamine hydrochloride (0.1 mL kg^−1^), medetomidine (Domtor^®^, Ecuphar Veterinaria, Barcelona, Spain; 0.05 mL kg^−1^), and butorphanol (Torbugesic^®^, Zoetis, Madrid, Spain; 0.1 mL kg^−1^) administered by means of intramuscular injection. Serum samples (2 mL) were taken from anesthetized trapped badgers by jugular venipuncture and collected in serum separation vacutainer tubes. Afterwards, badgers were euthanized with an overdose of sodium pentobarbital for post-mortem examination. The other 575 were killed in road traffic accidents and found by gamekeepers in the region. Serum samples (2 mL) from badgers killed in road traffic accidents were taken from the heart or thoracic cavity and collected in vacutainer tubes.

The location, sex, age class (subadult/adult), and weight were recorded for trapped and road-killed badgers. The entire group contained 301 males and 322 females, comprising 163 subadults and 460 adults based on body size and teeth. Sex and age class were undetermined for 50 individuals killed in road traffic accidents because of insufficient tissue availability. The numbers of animals analyzed per year were as follows: 2008: 21; 2009: 37; 2010: 68; 2011: 45; 2012: 20; 2013: 23; 2014: 43; 2015: 22; 2016: 92; 2017: 57; 2018: 63; 2019: 102; and 2020: 80. Trapping was not carried out in 2012–2015, 2017, or 2020, so all badgers studied in those years were killed in road traffic accidents. Badgers were preserved at 4 °C, and a complete post-mortem examination of each carcass was conducted at the laboratory in less than 24 h.

##### 2.3.1.1. Pathology

Serial sections (0.2 cm) were taken from the lungs and lymph nodes (LNs) of the 673 badgers submitted for necropsy for further macroscopic observation. Gross visible lesions found during necropsy were recorded. Tissue samples from the lungs and retropharyngeal, submandibular, tracheobronchial, mediastinal, hepatic, and mesenteric LNs were further analyzed by histopathology from the nine animals that showed gross lesions. Samples for histopathology were fixed in 10% neutral buffered formalin and processed using standard methods. Sections were cut to a thickness of 4 µm and stained with hematoxylin and eosin and by the Ziehl–Neelsen method for the detection of acid-fast bacteria.

##### 2.3.1.2. Bacteriology and Typing

Bacteriological and molecular studies were performed in 639 out of the 673 badgers (the remaining 34 animals were excluded due to insufficient tissue availability to make the pools). Pools (2 g) of lungs and mandibular, retropharyngeal, tracheobronchial, mediastinal, hepatic, and mesenteric LNs were frozen at −20 °C for no longer than two weeks and subsequently used to isolate potential bacteria as described previously [12]. Members of the MTC or the *Mycobacterium avium* complex (MAC) were isolated using the Mycobacteria Growth Indicator Tube (MGIT) liquid medium system, the Löwenstein–Jensen solid medium with sodium pyruvate, and Coletsos solid media. The samples were decontaminated using the BBL MycoPrep Becton Dickinson kit (BD Diagnostic Systems, Franklin Lakes, NJ, USA) and then incubated at 37 °C in an MGIT liquid medium for at least 6 weeks using the automated BACTEC MGIT 960 (BD Diagnostic Systems). Solid cultures were incubated at 37 °C for at least 10 weeks.

Quantitative PCR to identify MTC species was performed on culture isolates using the MTC forward primer 5′-TAGTGCATGCACCGAATTAGAACGT-3′, the MTC reverse primer 5′-CGAGTAGGTCATGGCTCCTCC-3′, and the TaqMan probe YY/BHQ 5′-AATCGCGTCGCCGGGAGC-3′, which amplifies a 184-bp fragment [22,23,24]. MTC isolates were characterized using DVR-spoligotyping after hybridizing biotin-labeled PCR products onto a home-made spoligotyping membrane (VISAVET, Madrid, Spain). Results were recorded in SB code followed by a field of four digits according to the *M. bovis* Spoligotype Database [23,24]. In order to confirm similarity between the isolates from cattle and badgers in the same area, MIRU-VNTR typing was performed as described previously [23] using the following nine VNTR markers: ETR-A, ETR-B, ETR-D, ETR-E, MIRU26, QUB11a, QUB11b, QUB26, and QUB3232. A badger was classified as positive if MTC was isolated. MAC isolates were identified as described previously [25].

##### 2.3.1.3. Serology (P22 ELISA Assay)

Serum samples from the 673 badgers were frozen at −20 °C before processing and tested using a previous validated in-house indirect P22 ELISA to detect antibodies against the MTC in badgers [26].

**Figure 1:**
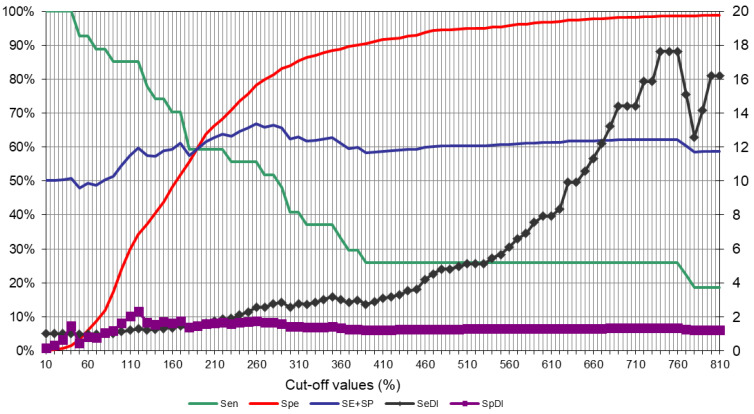
Definition of the P22 ELISA cut-off against a *Mycobacterium tuberculosis* complex (MTC) isolation reference. The graph includes data sera from positive and negative animals to bacteriological culture, as well as both trapped and road-killed animals. Sen: sensitivity; Spe: specificity; SE + SP: semi-sum or diagnostic value of sensitivity and specificity; SeDI: sensitivity discriminatory index (ratio of sensitivity to 1-specificity); SpDI: specificity discriminatory index (ratio of specificity to 1-sensitivity).

Briefly, plates were coated overnight with P22 in phosphate-buffered saline (PBS) at 4 °C and then blocked with PBS containing 5% powdered skim milk. After three washes with PBS containing 0.05% Tween-20, sera (diluted 1:100 in PBS-powdered skim milk) were added to duplicate wells and incubated for 60 min at 37 °C. Horseradish peroxidase-conjugated anti-badger CF2/HRPo IgG (100 μL) [26] was diluted to 1.5 µg/mL in PBS and added to the plates. Then plates were incubated at room temperature for 15 min in the dark with 3,3′,5,5′-tetramethylbenzidine substrate (Perbio Science, Helsingborg, Sweden). The reaction was stopped by adding 100 μL of 2 M H_2_SO_4_. Optical density (OD) was measured at 450 nm using an ELISA reader. As a negative control, serum from a TB-free Spanish badger was included in quadruplicate in every plate. As a positive control, serum from a Spanish badger experimentally infected with *M. bovis* was included in every plate. OD readings were expressed as an ELISA percentage (E%): E% = (mean OD/(2 × mean OD_negative control_)) × 100%. The cut-off was specifically defined for this study, since the previous P22 ELISA study in badgers suggested that the optimal cut-off (initially established between 100% and 150%) may depend on the epidemiological situation [26]. Indeed, when we applied the three cut-offs tested in that study (100%, 120%, and 150%), we found that sensitivity and specificity were too close to a random (50%) diagnostic value. Instead, we found that a cut-off of 260% gave 55.6% sensitivity and 80.4% specificity, corresponding to a moderate diagnostic value of 68% (Figure 1). In order to define the 260% cut-off, data sera from positive and negative animals (trapped and road-killed badgers) to bacteriological culture (MTC or MAC) were included.

#### 2.3.2. Cattle

Single intradermal tuberculin test data and bacterial isolation were available for cattle that were tested and culled as part of the Spanish National Program for the Eradication of Bovine TB until 2019 [2]. A total of 17,474 herds were studied, of which 4762 belonged to the western area, 8281 belonged to the center area, and 4431 belonged to the eastern area.

### 2.4. Statistical Analysis

An overall descriptive analysis of absolute frequencies was performed based of tables and chi squared tests with the SAS (Cary, NC, USA) Freq procedure. Differences in MTC isolation, P22 ELISA E% value, and positive frequency in badgers stratified by area, sex, age class, origin, or time were tested using a generalized linear model (GLM) in SAS with Tukey or Tukey–Kramer’s t (T or TK t) test for main effects level means comparisons. Differences with a *p* < 0.05 were considered significant. Potential associations between P22 ELISA values and TB prevalence in cattle during follow-up were tested using a Pearson correlation analysis with the SAS CORR procedure. Another GLM model was built to assess a potential association between the ability to isolate MTC or MAC from samples and the sample’s P22 ELISA E%.

## 3. Results

### 3.1. Badgers

#### 3.1.1. Gross and Microscopic Lesions

Gross lesions were observed in nine badgers from the eastern area, eight of which had been trapped and one of which had been killed in a road traffic accident. Lesions consisted of granulomatous areas of caseous necrosis and mineralization with diameters from 1 mm to 1 cm in submandibular, retropharyngeal, bronchial, or mediastinal LNs and lungs. Such lesions were also found in mesenteric LNs in two animals and in hepatic LNs in three animals. TB-like lesions were confirmed using histology, which revealed scarce, small granulomas with areas of central necrosis or mineralization. Ziehl–Neelsen staining revealed sparse acid-fast bacteria.

#### 3.1.2. Bacteriological Isolation and Typing

**Figure 2:**
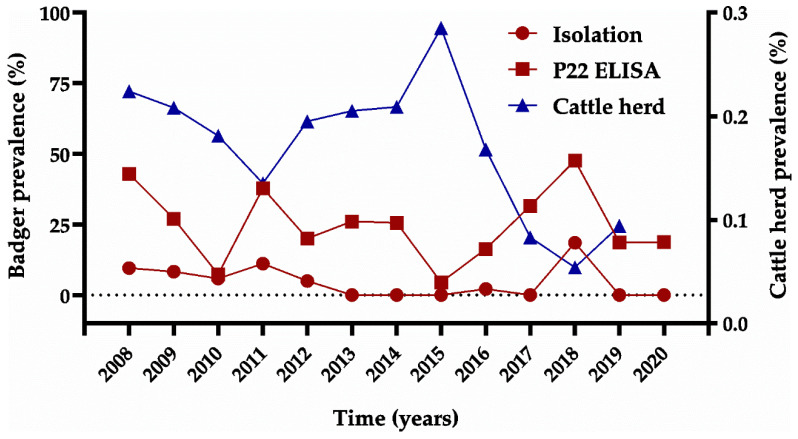
Prevalence of tuberculosis (TB) among badgers (red lines) and cattle (blue line) by year (2008–2020). Red circles refer to results based on bacterial isolation; red squares refer to results based on the P22 ELISA. TB prevalence based on single intradermal tuberculin test data and bacterial isolation was available for cattle herds as part of the Spanish National Program for the Eradication of Bovine TB [2].

**Figure 3:**
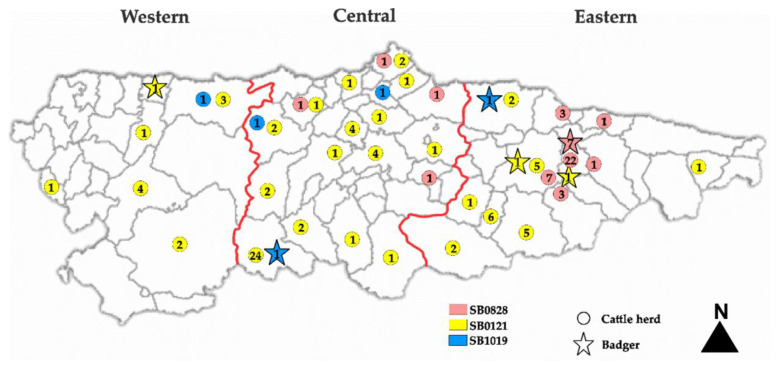
Shared *Mycobacterium bovis* spoligotypes (SB) identified in badgers and cattle herds in the three regions of Asturias (Atlantic Spain) from 2008 to 2020. The numbers within the color circles or stars indicate the number of badgers or cattle herds, respectively, positive to that SB during the 13-year follow-up. Red lines within the map establish the three regions of Asturias.

Of the 639 badgers, 27 (4.23%) were positive for MTC, and the distribution of positives across the years was as follows (Figure 2): 2008: 2; 2009: 3; 2010: 4; 2011: 5; 2012: 1; 2016: 1; 2018: 10; and 2019: 1. Isolates were identified as spoligotype SB0828 with VNTR profile 5-5-3-4-5-9-3-3-6 (*n* = 7), spoligotype SB0121 with VNTR profile 5-4-3-3-5-F-2-5-8 (*n* = 3), spoligotype SB0120 (*n* = 2), spoligotype SB01019 (*n* = 2), spoligotype SB1312 (*n* = 1), and spoligotype SB0329 (*n* = 1). Spoligotypes SB0828 and SB0121 identified in badgers were also isolated from cattle in the same areas (Figure 2) [24]. Spoligotype SB0828 with VNTR profile 5-5-3-4-5-9-3-3-6 was also identified in cattle, but spoligotype SB0121 in badgers (VNTR profile 5-4-3-3-5-F-2-5-8) was different, albeit related to that in cattle (VNTR profile 4-4-3-3-5-10-2-5-8). SB1019 was identified in badgers and cattle but in populations from different geographical areas (Figure 3). Neither spoligotype nor VNTR profiles could be determined from other badgers because of insufficient DNA.

**Figure 4:**
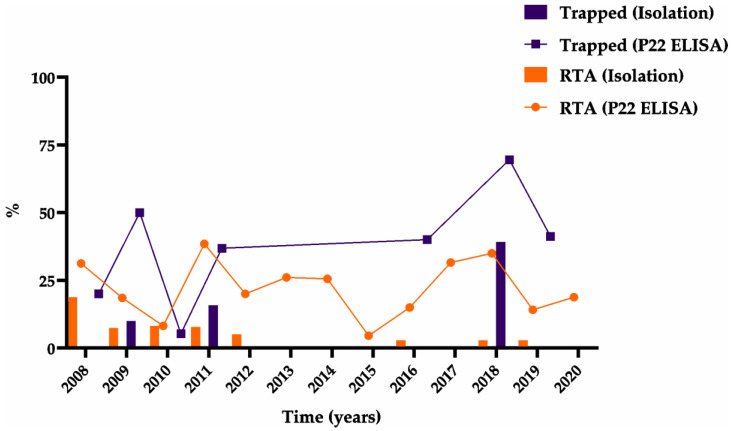
Prevalence of tuberculosis among trapped badgers (purple color) or badgers killed in road traffic accidents (RTA) (orange color) by year. The isolates are represented with columns for each of the two animal groups, and the lines represent seroprevalence. Circles refer to results based on P22 ELISA for the RTA, and the squares refer to results based on P22 ELISA for trapped animals. Note that trapping was not carried out in 2012–2015, 2017, or 2020, so all badgers studied in those years were RTA.

The rates of MTC positivity were 13.27% (13/98) among trapped badgers and 2.59% (14/541) (chi square = 23.37; *p* < 0.0001) among those killed in road traffic accidents (Figure 4, Supplementary Material 2: Trapped and RTA Badgers). The rate of positivity non-significantly varied with geographic region: 5 of 75 badgers (6.67%) from the western region were positive, compared to 4 of 209 (1.91%) from the central area and 18 of 355 (5.07%) from the eastern area. The rate of positivity was 3.81% (11/289) among males and 5.05% (16/317) among females. It was 4.40% (7/159) among subadults and 4.47% (20/447) among adults. Badgers with undetermined sex or age were all negative. No significant differences were found between areas (F = 1.78; *p* = 0.1695), sex (F = 1.12; *p* = 0.2898), or age class (F = 0.23; *p* = 0.6300) regarding MTC isolation. The only significant effects on isolation were year (F = 2.94; *p* = 0.0005) and origin (F = 6.01; *p* = 0.0145). We found significant differences in the P22 ELISA results between positive and negative animals in the bacteriological analyses (*p* < 0.0001).

**Figure 5:**
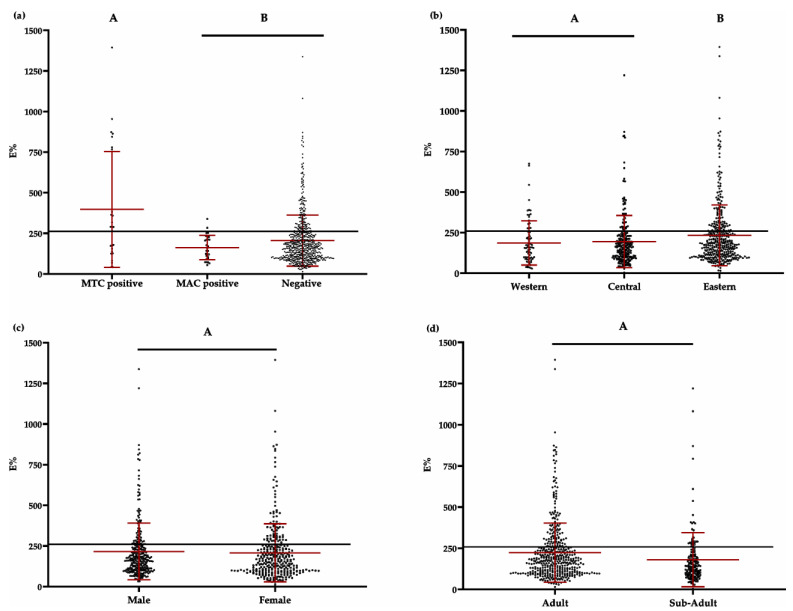
Proportions of badgers testing positive based on E% value in the P22 ELISA. (**a**) Positive badgers for *Mycobacterium tuberculosis* complex (MTC), positive for the *M. avium* complex (MAC), or negative for both complexes. (**b**) Positive badgers in western, central, and eastern parts of Asturias. (**c**) Male and female positive badgers. (**d**) Adult and sub-adult positive badgers. In all graphs, the horizontal black line represents the cut-off of 260% (see Section 2.3.1.3). The red lines represent means and standard deviations. Data connected with the same letter (A or B) are not significantly different based on Tukey–Kramer’s test (*p* > 0.05). Results from animals in which sex or age class were unknown are not included in panels c and d.

Of the 639 badgers, 28 (4.38%) were positive for MAC (Figure 5a): *Mycobacterium avium avium* (Maa, *n* = 14), *Mycobacterium avium hominissuis* (Mah, *n* = 8), or both (*n* = 1). Three animals positive for Maa were also positive by *M. bovis* isolation, and the spoligotypes were identified as SB0120 in two animals and SB0828 in the other. MAC isolates from five badgers could not be typed because of insufficient DNA. No differences were observed between the years 2008 and 2020 (4.76%, 5.56%, 8.82%, 4.45%, 0.00%, 0.00%, 4.65%, 4.55%, 2.17%, 0.00%, 5.56%, 4.12%, and 7.94%) (chi square = 11.70; *p* = 0.4699), origins (2.04% and 4.81% for trapped and road-killed, respectively) (chi square = 0.80; *p* = 0.3700), areas (2.67%, 5.74%, and 3.94% for western, central, and eastern, respectively) (chi square = 1.71; *p* = 0.4242), sex (3.46% and 5.68% for males and females, respectively) (chi square = 5.05; *p* = 0.0799), and age class (5.03% and 4.47% for subadults and adults, respectively) (chi square = 1.91; *p* = 0.3848). Badgers with undetermined sex or age class were all negative.

#### 3.1.3. Serological Assay (P22 ELISA)

A total of 160 badgers (23.77%) were positive in the P22 ELISA, and the distribution of positives by year was as follows (Figure 2): 2008: 9 (42.86%); 2009: 10 (27.03%); 2010: 5 (7.35%); 2011: 17 (37.78%); 2012: 4 (20.00%); 2013: 6 (26.09%); 2014: 11 (25.58%); 2015: 1 (4.55%); 2016: 15 (16.30%); 2017: 18 (31.58%); 2018: 30 (47.62%); 2019: 19 (18.63%); and 2020: 15 (18.75%). The rates of positivity based on P22 ELISA were 42.86% (42/98) among trapped badgers and 20.52% (118/575) among those killed in road traffic accidents.

The rate of seropositivity varied with geographic region as follows (Figure 5b): 17 of 77 badgers (22.08%) from the western region, 39 of 220 (17.73%) from the central area, and 104 of 376 (27.66%) from the eastern area were positive. The rate of seropositivity was 23.92% (72/301) among males, 22.05% (71/322) among females, and 34.00% (17/50) among animals whose sex was undetermined (Figure 5c). The rate of seropositivity was 17.79% (29/163) among subadults, 24.78% (114/460) among adults, and 34.00% (17/50) among badgers of underdetermined age class (Figure 5d).

Seropositivity significantly varied with year (F = 3.36; *p <* 0.0001), although a clear trend across years did not emerge. Seropositivity rates did not significantly differ (F = 1.93; *p* = 0.1465) between areas, although the frequency was slightly higher in the eastern area (37.04%) than in the central (30.94%) and western (29.44%) areas. However, mean E% did significantly differ according to geographical area (F = 3.91; *p* = 0.0204), with the eastern area (E% = 273.55) being higher than the western area (E% = 214.53; TK t = 2.73; *p* = 0.0179) but not than the central area (E% = 254.41; TK t = 1.31; *p* = 0.3881). The GLM identified origin (trapped or road-killed badgers) as the most influential factor on seropositivity (F = 15.86; *p* < 0.0001), with trapped animals being more positive (42.36%) than the road-killed ones (22.58%). Neither sex (F = 0.35; *p* = 0.5515) nor age class (F = 0.85; *p* = 0.3568) showed significant differences between categories (Figure 5d). Mean E% values were higher for badgers from which MTC was isolated (367.34) than for badgers from which MAC was isolated (162.35, TK t = 4.37; *p* < 0.0001) and badgers from which neither MTC nor MAC was isolated (204.91, TK t = 4.63; *p* < 0.0001). In turn, the mean E% values of culture-negative animals (neither MTC nor MAC was isolated) were higher (204.91) than those of MAC-positive animals (162.35), although they were non-significantly different (TK t = 1.30; *p* = 0.6884).

### 3.2. Cattle

During the study period, 362 cattle herds (2.07%) were positive for TB [2]: 84 (1.76%) were from the western area, 144 (1.74%) were from the central area, and 134 (3.04%) were from the eastern region. Prevalence remained below 1% throughout the period of study (Figure 2). The shared *M. bovis* spoligotypes identified in cattle and badgers from 2008 to 2020 in Asturias are shown in Figure 3.

**Figure 6:**
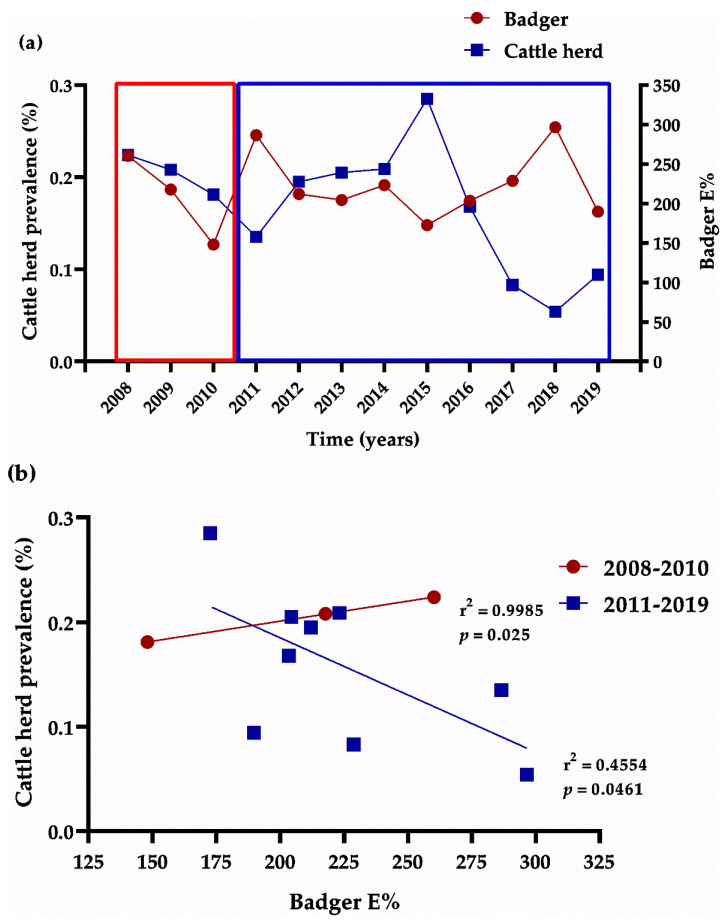
Correlation of tuberculosis (TB) prevalence in badgers and cattle herds in Asturias (Atlantic Spain). (**a**) Evolution of TB prevalence in badgers (red line) and cattle (blue line) by year. Prevalence in badgers was based on mean E% in the P22 ELISA. (**b**) Correlation between TB prevalence in cattle herds and mean E% in the P22 ELISA in badgers.

The prevalence of TB in cattle tended to negatively correlate with mean E% in the P22 ELISA in badgers, but the correlation was not significant (Pearson r; *p* = 0.489). In addition, the tendency inverted over time: it was positive between 2008 and 2010 and between 2012 to 2014, but it was negative for the rest of the period (Figure 6).

## 4. Discussion

This study examined the prevalence of TB in free-ranging Eurasian badgers in Spain for a 13-year period, which was longer than a previous seven-year study [12]. One study in England examined TB prevalence in badgers over 24 years [19], but only trapped animals were analyzed. In the present study, trapped animals and those killed in road traffic accidents were examined, with 4.23% badgers positive for MTC based on isolation and 23.77% badgers positive based on P22 ELISA. Such studies are essential for understanding pathogen transmission between species, particularly in endemic areas or hotspots, since such transmission can vary strongly from location to location [27].

P22 ELISA has provided high levels of sensitivity in previous studies in badgers [26]. Other available serological tests for diagnosing TB in badgers [28], e.g., the DPP Vet TB, the BrockTB STAT-PAK, and the chemiluminescent multiplex ELISA system have shown low-to-moderate sensitivity (from 30.77% to 58%) in naturally infected badgers [29,30,31,32,33]. P22 is mainly composed of MPB70 and MPB83, but there are other proteins shared with MAC or other non-tuberculous mycobacteria with a high sequence similarity to MPB70 and MPB83 [34]; that is why the cut-off is advisable to specifically define for each epidemiological situation [26]. We considered the cut-off of 260% adequate for the present study in order to avoid misclassification in an epidemiological context where MAC isolates were frequent. We found a big difference in TB prevalence using bacteriology or serology (4.32 vs. 23.77, respectively). The isolation of MTC has been considered as the gold standard method for the diagnosis of TB in wildlife [28], but sensitivity can be variable due to the lack of the active shedding of the microorganism from infected animals—hence the absence of mycobacteria in collected samples or when the number of viable microorganisms is low [35].

We detected TB-positive badgers in all parts of Asturias using both techniques, suggesting the widespread distribution of infection, mainly in trapped badgers from areas close to TB-positive farms. Our results were consistent with the idea that badgers are good hosts for TB; for example, TB-infected badgers living more than 1 km away from culled setts were detected even five years after the most recent outbreak in local cattle [36]. Indeed, TB transmission within badger subpopulations has been observed [37]. Given the widespread geographic distribution of positive badgers in our study, we suspect that badgers in Asturias have been infected on several occasions at different locations. We cannot exclude the possibility of transmission from cattle to badgers since both species shared the same or similar *M. bovis* genotypes, although this transmission is more likely when cattle have advanced disease [37] and such animals should be rare due to annual testing of all cattle in Asturias. Nevertheless, we observed a trend toward higher numbers of TB infections in cattle and badgers in the eastern part of the region, which supports the idea of cattle-to-badger transmission. This trend undoubtedly also reflects that most TB hotspots in Asturias lie in the eastern part, where several outbreaks of TB in cattle and badgers have been described, suggesting interspecies transmission and a high environmental contamination [12]. The presence of such hotspots may help explain why TB prevalence among trapped badgers in our study was more than two times the prevalence in road-killed badgers based on serology and more than four times the prevalence based on MTC isolation—trapped animals were captured in areas under active surveillance because of recent and ongoing outbreaks in cattle. Indeed, eight of the nine animals that showed gross lesions and whose infection was further confirmed by histopathology had been trapped. At the same time, we cannot exclude that at least some of the large difference in TB prevalence between the two groups of animals reflected the greater sensitivity of MTC detection in serum and tissue samples from trapped animals given that the quality of those samples may have been higher than the quality of samples from animals killed in road traffic accidents due to better preservation.

In a total geographical context, while TB prevalence among cattle herds has decreased in Asturias in recent years (reflecting the success of the official national eradication campaigns), TB prevalence among badgers has slightly increased. The increase in prevalence among badgers correlated with an increase in prevalence among cattle from 2008 to 2010 but with a decrease in prevalence among cattle from 2011 to 2019. This correlation was weaker regarding badger prevalence, as measured either by isolation or by P22 ELISA. Nevertheless, the number of positive badgers might have increased from 2016 to 2019 due to outbreaks in cattle, which itself was likely due to environmental contamination and indirect interspecies transmission [12]. This illustrates how badgers might likely transmit TB back to cattle in the future, highlighting the importance of understanding badger ecology for monitoring and controlling TB risk in cattle [12].

In our study, male badgers were marginally but not significantly more likely than female badgers to test positive for TB, which was different to studies in UK [38,39]. A study of 94 badgers killed in road traffic accidents in the UK found no sex difference in TB prevalence [40]. Studies have suggested that male badgers may transmit *M. bovis* to one another through biting during aggressive behavior [27,41]. We did not observe bite wounds in the present study, but this possibility should be explored in future work.

Adult badgers in our sample were more likely, although not significantly, to test seropositive than subadults. In our study, adult animals also showed higher E% in the P22 ELISA than subadults, probably reflecting the longer time available for animals to come into contact with *M. bovis*. The rates of MTC isolation did not significantly vary with badger age class, consistent with other studies in Europe [36,40]. Badgers in our study from which neither MAC nor MTC was isolated gave slightly higher E% in the P22 ELISA than badgers from which MAC was isolated, supporting the specificity of the assay. Our observation of intermediate E% for badgers from which neither MAC nor MTC was isolated suggests that some of these cases may have been false negatives because an immune response was apparent.

Of the 612 badgers that were TB-negative based on culture, 133 were seropositive based on the P22 ELISA, which means that the ELISA had a complementary sensitivity of 492.59% with respect to bacteriological isolation. Thus, taking the positives to either isolation or ELISA as reference, ELISA sensitivity was 84.57% compared to 15.43% for isolation. The sensitivity of culture tests might have been low because of poor sample condition, the technical limitations of the microbiological procedure, and the clinical stage of the badgers. In fact, only nine animals (a third of MTC-positive badgers) showed gross lesions, implying that most animals might have been in an early stage of infection or be MTC subclinical carriers, when MTC isolation is less sensitive [8,37]. Those early or latent stages, with infection confirmed by either immunology or bacteriological culture but with an absence of detectable pathology, are usual in badgers and have less risk of shedding greater numbers of mycobacteria than badgers in the late stages of disease [37]. We cannot exclude that other members of the MTC were present but not recovered by culture and cross-react in P22 ELISA, i.e., *Mycobacterium microti* infections have been reported in both wildlife [42,43] and domestic animals [44,45] in France, although the direct detection of that species on tissue samples was not included in this study. On the other hand, the relative specificity of each method with respect to the other remained much higher for isolation (97.45% vs. 78.27% for culture and P22 ELISA, respectively). In this regard, bacterial isolation remains the gold standard for the diagnosis of TB despite of methodological issues such as moderate sensitivity and long incubation times. Nevertheless, our results highlighted the P22 ELISA as a useful screening tool that is faster and more cost-effective and sensitive in the early or latent stages of disease than isolation for TB detection. Similarly, direct qPCR on tissue samples is also considered to be a sensitive and valuable alternative to culture that is faster than bacterial isolation [46]. Though qPCR can also detect non-viable mycobacteria, both techniques (P22 ELISA and qPCR) might be used in parallel in order to increase sensitivity and specificity of TB diagnosis in badgers.

## 5. Conclusions

We found that the TB status of badgers in Asturias during 2008–2020 was associated with the TB status of local cattle herds. Our results could not determine the direction of possible interspecies transmission, but they were consistent with the idea that the two hosts may exert infection pressure on each other. Adult badgers were more likely to be TB-positive than subadults. This study highlights the importance of monitoring this multi-host infection and disease in wildlife during epidemiological interventions in order to optimize outcomes under the One Health concept.

## Data Availability

The data published in this study are available in Supplementary Material 1: Raw Data and Supplementary Material 2: Trapped and RTA Badgers.

## Supplementary Materials

The following are available online at https://www.mdpi.com/article/10.3390/ani11051294/s1, Supplementary Material 1: Raw Data; Supplementary Material 2: Trapped and RTA Badgers.
